# Is there crosstalk between circadian clocks in plants and the rhizomicrobiome?

**DOI:** 10.1186/s12915-022-01443-8

**Published:** 2022-10-28

**Authors:** Xinming Xu, Antony N. Dodd

**Affiliations:** 1 Bacterial Interactions and Evolution Group, DTU Bioengineering, Technical University of Denmark, 2800 Lyngby, Denmark; 2 Department of Cell and Developmental Biology, John Innes Centre, Norwich Research Park, Norwich, NR4 7RU UK

**Keywords:** Circadian rhythms, Rhizomicrobiome, Microbial ecology, Plant-microbe interactions

## Abstract

Circadian clocks occur across the kingdoms of life, including some fungi and bacteria present in the root-associated soil known as the rhizosphere. Recent work from Amy Newman and colleagues, published in BMC Biology, has discovered that the circadian clock in Arabidopsis plants affects the rhythmicity of rhizosphere microbial communities This brings into play the exciting question of whether there is a bidirectional rhythmic interaction between plants and their rhizomicrobiome. Here, we discuss how the findings of Newman et al. suggest that soil microbiomes can have both self-sustained and plant-imposed rhythmicity, and the challenges of plant-microbiome circadian clock research.

## Commentary

Circadian rhythms are biological cycles that have a period of about 24 h, and persist in the absence of external cues. They provide a temporal structure to living organisms, aligning the phase of biological processes with the daily cycle of day and night. This is thought to provide a competitive advantage by ensuring that biological processes occur at the most appropriate time of day, and to capitalize upon 24 h fluctuations in the presence of environmental and endogenous resources or stresses. In recent research published in BMC Biology, Amy Newman and colleagues made the exciting discovery that the circadian clock in plants can influence circadian rhythms that occur in the composition of the rhizosphere microbial community [1].

**Fig. 1.**
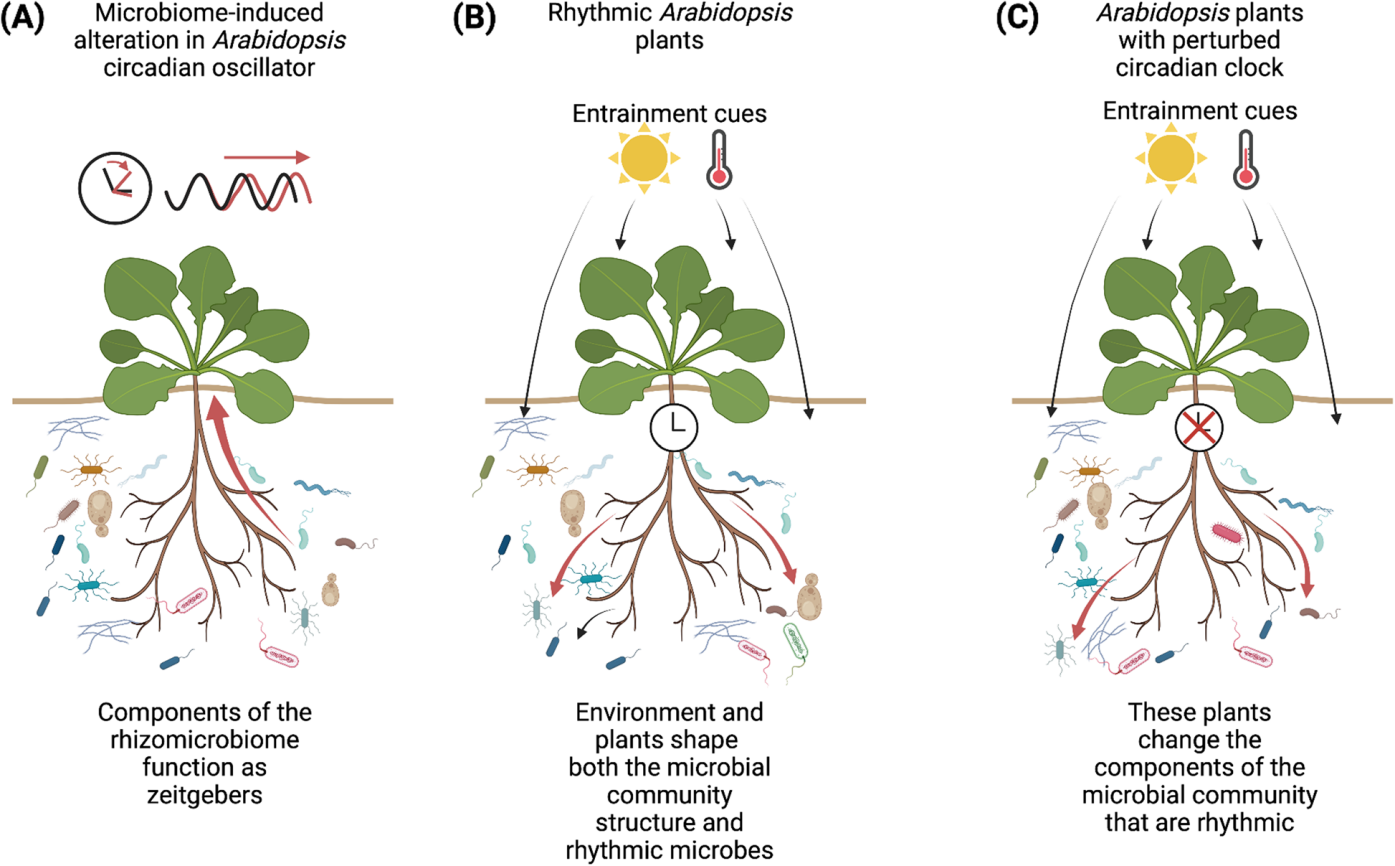
Potential interactions between circadian rhythms in plants and the rhizomicrobiome. **A** The rhizomicrobiome composition influences the period of the Arabidopsis circadian oscillator, suggesting that the rhizomicrobiome might function as a zeitgeber. **B** 24 h fluctuations in environmental conditions lead to entrainment of the Arabidopsis circadian clock, and also influence the rhizomicrobiome composition. The presence of an intact circadian oscillator in Arabidopsis influences the dynamics of the rhizomicrobiome composition. **C** Perturbation of the Arabidopsis circadian clock by misregulation of clock gene expression causes changes in the rhizomicrobiome composition, with changes occurring in which microbes are rhythmic

The competitive advantage provided by circadian clocks requires correct temporal alignment between the circadian clock and the environment [2]. This alignment is established by a variety of environmental variables- known as zeitgebers- that adjust the phase of the circadian oscillator, through the process of entrainment, so that it is appropriate for the environmental conditions. In plants, these environmental cues include light, temperature and metabolites. The rhizosphere microbial community also influences the functioning of the circadian clock in plants. For example, when the circadian period was compared for *Arabidopsis thaliana* (Arabidopsis) plants that were inoculated with a soil microbiome and an autoclaved microbiome, the circadian period of the plants with an intact microbiome differed from the manipulated microbiome, and the variation in circadian period between individuals was smaller [3]. This suggests that rhizomicrobiome components could have the potential to act as zeitgebers (Fig. 1A).

If components of the rhizomicrobiome can act as zeitgebers, this implies that there could be circadian rhythmicity within the rhizomicrobiome. In this context, there is evidence for circadian clocks in several bacteria. Amongst the prokaryotes, a considerable depth of mechanistic evidence for circadian rhythms has been obtained from photosynthetic bacteria, with a focus on using certain cyanobacteria as model organisms. For example, the unicellular marine cyanobacteria *Synechococcus* spp. is reported to have circadian rhythms of nitrogen fixation and photosynthesis, suggesting that this bacterium evolved a timekeeping system to confer fitness within a daily fluctuating environment [2]. The purple bacterium *Rhodospirillum rubrum* has a rhythmic pattern of hydrogenase activity that coordinates its energy metabolism according to the environmental conditions [4]. Although most purple bacteria do not have the self-sustained oscillations that are a defining characteristic of a circadian rhythm, it nevertheless implies that timekeeping programs can exist in a variety of bacteria.

Circadian rhythms are not restricted to photosynthetic bacteria, with fascinating examples of *circa*-24 h oscillations being reported in non-photosynthetic bacteria. The growth of human gastrointestinal bacterium *Klebsiella aerogenes* is sensitive to the neurohormone melatonin, and has a temperature-compensated, free-running circadian rhythm in swarming and motility [5]. It is reasoned that the clock of *K. aerogenes* might allow synchronization with the human circadian system [5]. Furthermore, Soriano et al. demonstrated how the light/dark cycles affect the growth of the plant-colonizing bacterium *Pseudomonas putida* [6]. When the cultures were spotted onto agar media with protein or extracellular polymeric substance-binding dyes, rhythmic bacterial growth could be detected both under 24 h light/dark cycles and constant darkness [6]. Another soil-dwelling rhizobacterium, *Bacillus subtilis*, has a circadian clock with canonical properties of circadian rhythms, and provides a powerful genetic and molecular model to unpack the mechanisms of circadian regulation in non-photosynthetic bacteria [7]. Apart from its various advantages for genetic manipulation, *B. subtilis* is one of the most thoroughly studied plant growth-promoting rhizobacterium, and has been identified from the rhizosphere of distinct plants. There is complex feedback between *B. subtilis* and plants, whereby plant root exudates serve as nutrient sources and signaling molecules, and *B. subtilis* possesses direct and indirect mechanisms to promote plant growth. This provides considerable experimental power to explore plant-microbiome clock regulations in a bidirectional system.

Circadian clock research is moving from single model systems to community-level studies. Zhao et al. tracked 24 h changes to microbial communities during rice cultivation, by collecting bulk soil and rhizosphere samples under light–dark cycles and constant dark regimes. Their results indicate that light exposure changes the microbial community structure, with co-occurrence networks suggesting that samples in the light had fewer vacant niches than in the dark [8]. Furthermore, when the rhizosphere microbiome was compared for wild type, short- and long circadian period Arabidopsis plants, the rhizosphere community harboured by the short period mutants had lower species richness and evenness compared with wild type plants [9]. In addition, when the wild type plants were cultivated on soil that was pre-conditioned by growing wild type or circadian period mutant Arabidopsis, plant growth was reduced in the presence of the clock-mutant preconditioned microbiomes [9]. This suggests that plant circadian oscillator components, or properties of the rhythm such as the period or phase, affect the microbiome structure. This might occur through changes in plant development or root exudation. Together, these studies identify 24 h fluctuations in rhizosphere structure that are influenced by plants, opening further questions about roles for circadian clocks in this interaction.

Newman et al. [1] made an exciting contribution to this field by demonstrating that there are rhythms in the rhizosphere microbiome community structure that occur under both cycles of light and dark, and under constant conditions (Fig. 1B). Perturbation of the Arabidopsis circadian oscillator by overexpression of the clock component LATE ELONGATED HYPOCOTYL (LHY) (LHY-ox, which causes arrhythmia under constant conditions), and also a mutant of the *LHY* gene (*lhy*-11), caused alterations in the rhythmicity of community structure of the rhizomicrobiome (Fig. 1C). They found that the different plant genotypes recruited different rhizomicrobiome communities, implying selection for niche-adapted organisms. Some operational taxonomic units (OTUs) that were rhythmic in the presence of wild type plants became arrhythmic in the presence of LHY-ox or the *lhy*-11 mutant, and some OTUs that are arrhythmic in the presence of wild type plants assumed rhythmicity in the presence of these plants with perturbed circadian clocks (Fig. 1B, C). The presence of rhythmicity of many bacterial and fungal OTUs required a rhythmic plant circadian clock, yet some individual OTUs appeared to have self-sustained endogenous circadian timing.

Quantification of plant circadian clock parameters- using methods such as luciferase imaging- is comparatively straightforward compared with the detection of rhythmicity in microbial communities. How can specific circadian signals be detected within microbial communities? Sometimes, researchers are interested in taxa that change between the light and dark periods of a light–dark cycle, and use methods such as differential analysis to identify the indicator taxa. It is important to consider that microbiome data are often relative (proportional) abundances within the entire microbiome, thus changes in absolute abundance of single taxa can alter the apparent relative abundance of all taxa. Therefore, 24 h oscillations of the relative abundance of one highly abundant taxa could lead to variations in the proportional signal for other low relative-abundance taxa, producing false-positive detection of rhythmicity of the low abundance taxa. This illustrates how the monitoring of circadian rhythmicity within complex microbiomes presents interesting experimental and analytical challenges. Whilst the circadian clock is known to provide a competitive advantage to plants and certain cyanobacteria, the nature of any fitness advantage to individual species within a complex microbiome is less clear. It would be fascinating to know whether rhizomicrobiota gain fitness advantages through anticipation of 24 h environmental changes cycles, and perhaps occupy specific temporal niches within the 24 h cycle [10]. Understanding such implications of the work of Newman et al. might require future development of methodology and data types to bridge together the investigation of circadian programs at the plant and microbiome scales.

Taken together, the findings of Newman et al. are important for a variety of reasons. Firstly, they could suggest that there is signaling of circadian timing information from plants to their respective microbiomes. Furthermore, the existence of rhythmic components of the microbiome in the presence of arrhythmic plants supports the notion that there might be self-sustained circadian rhythmicity in the rhizomicrobiome composition. This is plausible, given that circadian clocks occur in fungi, nematodes, cyanobacteria and some non-photosynthetic bacteria [5, 7]. Finally, the emergence of rhythmicity of some microbes in the presence of arrhythmic plants, when those microbes are arrhythmic in the presence of wild type plants, suggests that 24 h oscillations of some soil microbes is conditional upon their environments. This is consistent with evidence that circadian rhythms in *B. subtilis* are conditional upon certain nutrient media types, and only when biofilm forms [7]. This emerging body of literature suggests that the community is embarking upon a fascinating scientific journey into the ecology of circadian rhythms.

## Data Availability

Not applicable.
